# Development and optimization of amphiphilic self-assembly into nanostructured liquid crystals for transdermal delivery of an antidiabetic SGLT2 inhibitor

**DOI:** 10.1080/10717544.2022.2144546

**Published:** 2022-11-15

**Authors:** Nancy M. Lotfy, Mohammed Abdallah Ahmed, Nada M. El Hoffy, Ehab R. Bendas, Nadia M. Morsi

**Affiliations:** a Future Factory for Industrial Training, Faculty of Pharmacy, Future University in Egypt, Cairo, Egypt; b Department of Pharmaceutics and Industrial Pharmacy, Faculty of Pharmacy, Cairo University, Egypt; c Department of Pharmaceutics and Pharmaceutical Technology, Faculty of Pharmacy, Future University in Egypt, Cairo, Egypt; d Department of Pharmacy Practice and Clinical Pharmacy, Faculty of Pharmacy, Future University in Egypt, Cairo, Egypt

**Keywords:** Canagliflozin, transdermal, liquid crystals, finite dose permeation, poloxamer, Monoolein

## Abstract

The anti-hyperglycemic sodium glucose co-transporter 2 inhibitor Canagliflozin (CFZ) represents a recent antihyperglycemic modality, yet it suffers from low oral bioavailability. The current work aims to formulate CFZ-loaded transdermal nanostructured liquid crystal gel matrix (NLCG) to improve its therapeutic efficiency. Pre-formulation study included the construction of pseudoternary phase diagrams to explore the effect of two conventional amphiphiles against amphiphilic tri-block copolymer in the formulation of NLCG. The influence of different co-solvents was also investigated with the use of monooleine as the oil. Physical characterization, morphological examination and skin permeation were performed for the optimized formulations. The formula of choice was further investigated for skin irritation and chemical stability. Pharmacodynamic evaluation of the successful formula was conducted on hyperglycemic as well as normoglycemic mice. In addition, oral glucose tolerance test was conducted. Results revealed the supremacy of Poloxamer for stabilizing and maximizing liquid crystal gel (LCG) area percentage that reached up to 12.6%. CFZ-NLCG2 isotropic formula showed the highest permeation parameters; maximum flux value of 7460 μg/cm^2^ h and *Q*_24_ of 5327 μg/cm^2^. Pharmacodynamic evaluation revealed the superiority of the antihyperglycemic activity of CFZ-NLCG2 in fasting mice and its equivalence in the oral glucose tolerance test (OGTT) compared to the oral one. The obtained results confirmed the success of CFZ-NLCG2 in the transdermal delivery of CFZ in therapeutically effective concentration compared to the oral route, bypassing first pass effect; in addition, eliminates the possible gastrointestinal side effects related to the inhibition of intestinal sodium glucose co-transporter (SGLT) and maximizes its selectivity to the desired inhibition of renal SGLT.

## Graphical Abstract

**Figure:**
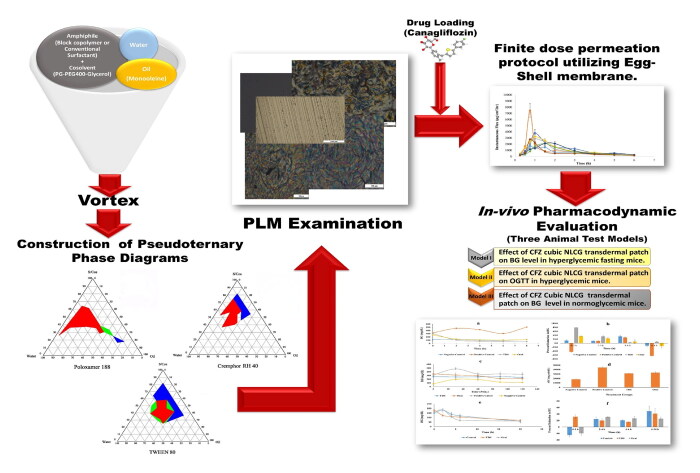

## 1. Introduction

One of the most recent oral antidiabetic drug classes is sodium glucose co-transporter 2 (SGLT2) inhibitors known as Gliflozins. Generally, SGLTs are glucose transporter proteins expressed mainly in the mucosa of the small intestine (SGLT1) and the proximal tubules of the kidney (SGLT1 and SGLT2). In renal tubules, SGLT2 is responsible for the majority of filtered glucose reabsorption. Inhibition of intestinal SGLT1 reduces glucose absorption and augments the release of gastrointestinal incretins, resulting in gastrointestinal complications, including severe diarrhea. On the other hand, SGLT2 inhibition reduces glucose reabsorption in the renal tubule, leading to increased glucose excretion (Hsia et al., 2017; McCrimmon & Henry, 2018; Padda et al., 2022). Inhibition of SGLT2 represents a novel insulin independent mechanism of action and is extremely beneficial in the treatment of patients whose pancreatic function is impaired or completely lost (Polidori et al., 2014). Canagliflozin (CFZ) was the first SGLT 2 inhibitor approved by the FDA for the treatment of type II diabetes (T2DB). Presently, the FDA approved the drug for both the prevention of cardiovascular system events in patients with diabetes and the reduction of the risk of end-stage kidney disease (Nespoux & Vallon, 2020; PubChem, 2021; Tian et al., 2021; Vallon & Verma, 2021; Vaduganathan et al., 2022).

CFZ is absorbed rapidly after oral administration, peak plasma concentration occurs within 1 to 2 h. The mean half-life is 10.6 and 13.1 h for the 100 mg and 300 mg drug dose respectively, hence a once daily dose is recommended (Rosenthal et al., 2015). The drug is extensively metabolized by glucuronidation leading to an oral bioavailability of 65%. The drug is extensively bound (98.3%–99.2%) to plasma proteins, particularly to human serum albumin. The mean steady-state volume of distribution following intravenous administration in healthy participants is 83.5 L. Approximately 60% and 33% of the administered dose is excreted in the feces and urine, respectively. The mean systemic clearance of canagliflozin after an intravenous infusion was reported to be 12.2 L/h (Devineni && Polidori, 2015). Several polymorphic forms of CFZ have been reported, among them, crystalline form hemihydrate and monohydrate can be found as the more frequently abundant polymorphs. The solubility of canagliflozin hemihydrate in water is 46.4 μg/mL. The drug has a partition coefficient (log*P*) of 3.44 and belongs to class 4 in biopharmaceutical classification (PubChem, 2021; Zhu et al., 2021). At high doses (>200 mg), the luminal concentration of canagliflozin is found to be high enough to inhibit intestinal SGLT1 and the drug is reported as a low-potency inhibitor of SGLT1 (Rosenthal et al., 2015; Triplitt & Cornell, 2015).

In the last decade, transdermal delivery systems (TDS) attracted researchers as a promising dosage form for antidiabetic agents (Ng & Gupta, 2020). Many of the side effects of oral antidiabetics could be eliminated using TDS. Delivering drug through skin enhances drug bioavailability by bypassing the first pass effect. Also, drug food interaction and drug gastrointestinal tract (GIT) side effects like diarrhea and gastric irritation are all avoided. In addition, the effect of pH and gastric emptying rate on the drug is excluded. In contrary to the pulsed drug release in case of oral formulation, TDS offer a controlled drug release over a predetermined period of time, this maintenance of steady-state absorption potentiates the drug therapeutic efficiency (Jeong et al., 2021). In geriatric medicine, transdermal devices are ideal for patients who suffer from difficulty in swallowing and who are not reliable in taking oral medication, elderly patients also may suffer from GITs problems that exacerbate adequate drug absorption. Moreover, transdermal delivery (TD) allows immediate elimination of the drug when needed as in the case of hypoglycemia by simply removing the patch. However, the major challenge in TDS is to overcome the barrier function of the stratum corneum which is considered the rate-limiting step in drug permeation (Jeong et al., 2021).

Forthcoming, ordered nanostructured amphiphilic based liquid crystal (LC) is anticipated to constitute a major share in the field of nanoscale soft matter technology as a consequence of its unsurpassed prospects in material templating and designs (Negrini & Mezzenga, 2012; Rajak et al., 2019). Formulation of drug in a lyotropic liquid crystal (LLC) matrix for TD is a very promising approach that interests many scientists in the last years owing to its high viscosity, and strong bioadhesive properties. Moreover, the similarity between the internal structure of the LLC drug matrix and the intercellular stratum corneum (SC) lipid structure is the foremost LC property that make it irreplaceable in enhancing drug skin penetration (Silvestrini et al., 2020). In addition, LLC are thermodynamically stable, hence they are easy to prepare as they are formed spontaneously and exhibit extraordinary loading capacity for both hydrophilic and lipophilic drug molecules. Protection of loaded drug molecules from hydrolysis and enzymatic degradation is another paramount criterion in selecting LLC as drug matrix for pharmaceutical compounds (Kim et al., 2015; Silvestrini et al., 2020; Saadat et al., 2021; Chountoulesi et al., 2022). However, when it comes to the mass production, LLC suffer from challenging processability that arise from the extremely high viscosity and stiffness in most applied systems. These poor handling and mechanical properties minimize their suitability in many applications and set barriers to their commercialization. Approaches to overcome these difficulties include the use of a flowable precursor or dispersing the bulk phase into nanoparticles.

Consequently, investigations are still needed for the utilization of bulk phase LLC as a scalable delivery system (Guo et al., 2010; Kim et al., 2015, Saadat et al., 2021; Türeli & Türeli, 2020). This could be attained by judicious choice of the composing materials and their concentrations to precisely customize a LLC system with the desirable characters for further application.

LLC exhibited various mesophases according to the internal architecture acquired by the self-assembled amphiphile, the most common mesophases are the anisotropic lamellar and hexagonal (type I and II) and the isotropic cubic (type I and II). Amongst all types, the lamellar, inverse cubic and inverse hexagonal attained great potential in the area of pharmaceutical delivery systems. Generally, LLC nanostructures are formed as a result of the interaction between oil, water and amphiphile. Such structures are tunable by the addition of other types of additives (Song et al., 2017).

Monoolein (MO) is an amphiphilic unique lipid that has been known as the magic lipid regarding its phase behavior in LCs. In aqueous solution, it can self-assemble into different LC phases according to the variation in the conditions of temperature and composition. In addition, it is biodegradable, biocompatible, bioadhessive and can accommodate a wide range of multiple molecular sizes of hydrophilic and hydrophobic drugs. Moreover, it has a proven safety profile.

Co-solvents are frequently used in pharmaceutical formulations and industry because of their moderate polarity that aids in drug solubilization. In addition, their use in LLC could modulate their mesomorphic structures as well as the handling and mechanical performance of the formula.

The presented work aimed at the development of a novel liquid-crystal based drug delivery system for the successful transdermal delivery of CFZ to bypass drug first pass effect and augment its selectivity to its target receptors. The LCs were prepared using simple mixing method. The obtained formulations were characterized in terms of visual inspection, morphological study, pH determination, rheological behavior, permeation study and accelerated stability testing for physical parameters. The optimized formulation was further investigated for its chemical stability and biological performance regarding skin irritation as well as its antihyperglycemic activity utilizing both the hyperglycemic as well as the normoglycemic mice models.

## 2. Materials and methods

### 2.1. Materials

Canagliflozin hemihydrate was obtained from Metrochem API private limited, India. Monoolein, streptozotocin (STZ) and acetonitrile HPLC grade were purchased from Sigma-Aldrich, USA. Polyethylene glycol 400 and Cremophor RH 40 were acquired from CISME, Italy. Ethanol, propylene glycol and glycerin were purchased from Chemajet, Egypt. Tween 80, sodium chloride, potassium dihydrogen phosphate, disodium hydrogen phosphate, sodium hydroxide, hydrochloric acid, hydrogen peroxide 30%, formaldehyde solution 38%, citric acid and sodium citrate were bought from Nasr Co, Egypt. Poloxamer188 was purchased from Alfa Aesar, USA. Orthophosphoric acid HPLC grade was obtained from Scharlau, Spain. Hydroxypropylmethyl cellulose (HPMC E5) was acquired from Shandong, China. Glucose solution 10% was bought from Autsoka, Egypt. Sucrose was purchased from ESIIC, Egypt.

### 2.2. Methodology

#### 2.2.1. Construction of pseudoternary phase diagram

Pseudoternary phase diagrams (PTPDs) were constructed for determination of the range of concentrations of the used components that can lead to the successful formation of nanostructured liquid crystal gel (NLCG) matrix. Graphs were drawn utilizing AutoCAD 2014, USA software. The used components were MO as oil, three different surfactants, namely; Tween 80, Cremophor RH 40 and Poloxamer 188. The effect of the presence of different co-solvents at three different mixing ratios (Km ratio) of surfactant to the co-solvent (S/Cos) was also investigated. The used co-solvents were propylene glycol (PG), glycerol, and polyethylene glycol (PEG 400) at *K*_m_ ratios of 4:1, 2:1 and 1:0. Aliquots of surfactant/co-solvent blend with the different *K*_m_ ratios were mixed with oil to give (surfactant/co-solvent):(oil) weight ratio of 9:1, 8:2, 7:3, 6:4, 5:5, 4:5, 3:7, 2:8 and 1:9. These mixtures of surfactant/co-solvent and oil were mixed by vortex with the aid of gentle heat to melt any solid constituents until homogeneous dispersion was obtained. These dispersions were then titrated with water at an amount of 0.1 g under vortex every 24 h to allow them to equilibrate. The mixtures were then assessed by visual inspection for transparency and consistency.

Percent of LCG area and the maximum water solubilized ratio (%*W*) were used as assessment criteria to choose the best NLCG systems for drug loading.

#### 2.2.2. Preparation of CFZ loaded NLCG formulae

Based on the obtained phase diagrams, for each surfactant type, the optimum (surfactant/co-solvent):(oil) ratio that could incorporate the largest amount of water in the NLCG area were chosen for further drug loading. Based on the same criteria, for each co-solvent type containing system, the type of co-solvent with the optimum *K*_m_ ratio that could give the largest NLCG area were also selected, systems which fail to form any NLCG area were excluded.

Drug (0.5–5 wt/wt%) was preliminary loaded with different concentrations at the selected systems to determine the maximum drug concentration which could be incorporated without affecting the consistency and clarity of the formed NLCG.

#### 2.2.3. Characterization of CFZ-loaded NLCG formulae

##### 2.2.3.1. Morphological examination

To identify the liquid crystal mesophase structure of the drug loaded formulae, a drop of the sample was applied on a glass slide and covered with a coverslip then investigated under the cross polarized light microscopy (PLM) (Nikon Optiphot-POL, Japan).

##### 2.2.3.2. Measurement of pH

The pH of the selected formulae was determined using a pH meter (Jenway, UK) after being diluted with water to10%.

##### 2.2.3.3. *In vitro* permeation study

*In vitro* permeation finite dose regimen was applied using eggshell membrane. The study was conducted using Franz diffusion cell (Hanson Research, MicroettePlus, USA). Sink condition was first guaranteed by dissolving ten times the amount of the applied drug dose in the specified volume of the receptor medium; 7 mL of 50% ethanolic phosphate buffer saline pH 7.4 (PBS 7.4 USA) (Selzer et al., 2013).

Eggshell membrane was mounted in the apparatus with effective permeation area of 1.76 cm^2^. Membrane preparation was carried out by submerging the entire egg in 5 M HCl to dissolve the outer solid shell, the egg was then evacuated from its content (Haigh & Smith, 1994; Olivella et al., 2006), the membrane was rinsed by distilled water then kept soaked in 50% ethanolic (PBS 7.4) for an hour before starting the experiment. An amount of 0.3 g of 4% of medicated NLCG formulae were applied to the membrane, which corresponds to a finite dose equal to 7 mg/cm^2^. The receptor medium was kept continuously stirring at 600 rpm during the time of the experiment and the temperature of the Franz system was adjusted to be 37 °C. Aliquots of 2 mL sample were withdrawn from the receptor medium at 0.25, 0.5, 0.75, 1, 1.5, 2, 3, 4, 5, 6, 8, 10, 12, 16, 20 and 24 h time intervals. Withdrawn samples were compensated by an equal volume of the receptor medium. Each sample was subjected, after suitable dilution, to spectrophotometric analysis at *λ*_max_ of 290 nm by a validated method of analysis (Nirali et al., 2015). Calibration curves were previously constructed for determination of the absorbance constant, all test samples were run against blank under the same experimental conditions using the corresponding base without drug. The cumulative amount of the drug permeated (*Qs*) through the membrane in the receptor compartment at the n^th^ sampling was estimated by the equation:

(1) $$Qs=\frac{V}{S}.\;Cn+\sum_{n-i}^{n-1}\frac{Vi}{S}.Ci\;$$

where *Cn* is the drug concentration in the receptor solution at the *n*^th^ sampling time, *Ci* is the drug concentration of the sample, and *V* and *Vi* are the volumes of the receiver solution and the withdrawn sample, respectively. *S* is the effective diffusion area. The cumulative amount of CFZ permeated was plotted as a function of time. Instantaneous flux against time curve was obtained by plotting the amount of drug permeated per unit time against time. From the two curves, the permeation parameters (*Q*_24_), the maximum flux (*J*_max_) and the time required to reach this flux (*T*_max_) were obtained (Lau & Ng, 2017). These permeation parameters were further used as assessment criteria for the selection of the most promising formula for the *in vivo* study.

##### 2.2.3.4. Rheological study

Viscosity of the prepared formulae was determined using Brookfield DV3T Viscometer, USA, with spindle CP-52. The rheological behavior of each formula was evaluated by plotting the shear rate against the obtained shear stress values, viscosity values of each drug loaded formula at minimum shear rate (SR) was also assessed.

##### 2.2.3.5. Accelerated stability study for physical parameters

All formulae were stored at ambient conditions for six months and checked for physical stability. Formulae that showed physical stability were subjected to accelerated stability study for physical parameters by storing for three months in stability cabinet under conditions of 40 °C temperature and 75% relative humidity. The formulae were then re-evaluated for their physical, rheological and drug permeation parameters through eggshell membrane.

#### 2.2.4. Choosing the optimized CFZ-NLCGs

Based on the obtained results, the desirability function was utilized for choosing the optimum formulation for further investigations. The criteria of choice was based on the suitability of the permeation parameters; maximization of *J*_ss_ and *Q*_24_ and minimization of *T*_max_.

#### 2.2.5. Characterization of the optimized CFZ-NLCG

##### 2.2.5.1. Chemical stability study of the optimized CFZ-NLCG

The formula of choice was further analyzed for chemical stability by a slightly modified validated reversed phase RP HPLC stability indicating method (Suneethal & Sharmila, 2015). The method was revalidated for linearity, accuracy, precision, robustness, selectivity and specificity. Forced degradation was also pursued under different stress conditions; namely, acid hydrolysis, alkali hydrolysis, oxidation and thermal degradation. Such conditions were attained by adding of 5 mL of 2 N sodium hydroxide, 2 N HCl and 20% H_2_O_2_ to 1 mL of the drug sample, and reflux for 30 min at 60 °C. Whereas, thermal degradation was done by heating the drug solution in an oven at 105 °C for 6 h. The working steps were carried out in isocratic mode using (Agilent-1260 Infinity, USA) HPLC apparatus, 0.1% orthophosphoric buffer:acetonitrile (53:47) were used as a mobile phase, C18 Hypersil BDS column (100 mm × 4.6 mm, 5 μ) was used. Column temperature was adjusted at 30 °C. Injection volume was 10 µL and flow rate was maintained at 0.85 mL/min. The measurements were taken at retention time of 11.3 min and 290 nm wavelength using photo diode array detector. Calibration curves of CFZ in the mobile phase solution were constructed.

##### 2.2.5.2. *In vivo* pharmacodynamic evaluation of the optimized CFZ-NLCG formula

###### 2.2.5.2.1. Animals

 Male albino mice 8 weeks age, weighing 30–35 g and male Wistar albino rats weighing 200–225 g were obtained from the animal house of Pharmacological Department, Future University in Egypt (FUE). Animals were kept in appropriate numbers in plastic cages and housed under conventional conditions with controlled temperature, humidity and natural light/dark cycles. Animals were acclimatized at these conditions for 14 days before starting the experiments and were provided with water and standard commercial diet ad libitum. All experimental procedures were approved by the research ethics committee of Faculty of Pharmacy, Future University in Egypt (PT: REC-FPSPI-11/73).

###### 2.2.5.2.2. Application of CFZ-NLCG transdermal formula 

A polyethylene membrane was fixed to the adhesive side of a surgical tape and a plastic ring with an area of 0.2 cm^2^ was mounted and tightly fixed to the polyethylene layer. An accurately weighed amount of the optimized CFZ-NLCG formula was added inside the area of the ring to be applied to the shaved dorsal area of the animal.

###### 2.2.5.2.3. Induction of hyperglycemia 

To induce hyperglycemia, the overnight fasted mice were injected with streptozotocin (STZ) intraperitoneally at a dose of 200 mg/kg after a pilot study. STZ was freshly prepared as 50 mM in cold citrate buffer (pH 4.5) to a final concentration of 20 mg/mL. 10% sucrose solution was given to mice via oral gavage to avoid hypoglycemia that may occur during the first 24 h. Blood samples were collected after 72 h, from the tail vein of fasting mice to assess the fasting blood glucose (FBG) levels (ACCU-CHEK Active, Roche, Germany). Mice with FBG > 250 mg/dL were considered hyperglycemic (Furman, 2021).

###### 2.2.5.2.4. Experimental design and test models

 Three experimental test models were conducted. In all models, the dorsal area of all animal groups was shaved by electric clipper one day before the experiment. A concomitant plain transdermal patch applied to the oral and the control animal groups. The test models were designed and conducted as follows:

*Model 1: Effect of CZF NLCG transdermal patch on blood glucose level in hyperglycemic fasting mice*

Animals were divided into four groups, hyperglycemic mice were randomly allocated to 3 groups, and one group of normoglycemic mice, each group consisted of six mice (*n* = 6). All mice were fasted overnight with free access to water only.

Group I: control of normoglycemic mice.

Group II: control of hyperglycemic mice.

Group III: Hyperglycemic mice received oral canagliflozin at a dose of 10 mg/kg, per oral, suspended in 0.5% HPMC E5 solution, the suspension was given by oral gavage.

Group IV: Hyperglycemic mice received the optimized CFZ-NLCG transdermal formula (at a dose of (10 mg/kg).

Blood samples were gathered at time intervals of 0, 2, 4, 6 and 8 h and the percent reduction in blood glucose (BG) levels were also calculated for each interval.

*Model 2: Effect of CZF-NLCG transdermal formula on oral glucose tolerance test (OGTT) in hyperglycemic mice*

Fasted animals were divided into four groups and received oral and transdermal treatment exactly as in the previous test model. After 30 min of drug administration, glucose solution was administered orally at a dose of 2 g/kg using oral gavage to all groups. Blood samples were obtained from tail vein for measuring BG levels at sampling intervals of 0, 30, 60, 90 and 120 min after glucose loading. The area under the curve (AUC) for the BG concentrations was calculated for each group using GraphPad Prism 7.03 software, USA (Okoduwa et al., 2017).

*Model 3: Effect of CZF-NLCG transdermal formula on blood glucose levels in normoglycemic mice*

Normoglycemic mice were maintained with free access to food and water overnight. At the start of the experiment, animals were deprived of food after drug administration (Tsuneki et al., 2002). The animals were divided into three groups as follows:

Group I: control of normoglycemic mice.

Group II: normoglycemic mice received oral canagliflozin at a dose of 10 mg/kg, per oral, suspended in 0.5% HPMC E5 solution, the suspension was given by oral gavage.

Group III: normoglycemic mice received the optimized CFZ-NLCG transdermal formula (at a dose of (10 mg/kg). Blood samples were taken from tail vein for determination of BG levels at time intervals of 0, 2, 4, 6 and 20 h and the percent reduction in BG levels were calculated for each interval.

##### 2.2.5.3. *In vivo* skin irritation test

The dorsal hair of the Wistar rats was removed by an electric clipper one day before the experiment. The rats were randomly divided into three groups each consists of three animals, group I served as the control and received no treatment, group II received 0.8% vol/vol aqueous formalin solution on the dorsal area as a standard irritant and group III was the test group in which the rats received the optimized CFZ-NLCG through application of approximately 0.5 g of the formula to the dorsal area of the skin then covered by a gauze which held in place by the aid of surgical adhesive tape (Patel & Gupta, 2013). After 24, 48 and 72 h, the site was examined for skin irritation. The application sites were evaluated visually for erythema and edema and scored as 0 for no erythema and edema, 1 for very slight erythema or edema, 2 for well-defined erythema and slight edema (barely perceptible), 3 for moderate to severe erythema and moderate edema (edges of area well-defined by definite raising) and 4 for severe erythema (beef redness to scar formation) and severe edema (extended beyond the area of exposure).

The score of primary irritation (SPI) was calculated for each rat according to Eq. 2 as follows (Patel & Gupta, 2013):

(2) $$\mathrm{SPI}\;=\;\frac{\sum \text{Erythema and Edema grade at }24,\;48\;\mathrm{and}72\;\mathrm{hrs}.}{\text{Number of observations}}\;$$

The difference between the summations of SPI scores of three animals from test-treated rats and control rats were calculated and used for determination of primary irritation index (PII) by Eq. 3.

(3) $$\mathrm{PII}\;=\frac{\text{ƩSPI }(\mathrm{Test})\;–\text{ ƩSPI }(\mathrm{Control})}{\text{Number of animals}}\;$$

The extent of irritation was classified according to the PII as negligible for value of (0–0.4), slight for value of (0.5–1.9), moderate for value of (2–4.9) or severe for value ranges from 5 to 8.

#### 2.2.6. Statistical analysis

All data were treated statistically by applying two-way ANOVA. Significance difference of data was accepted at *p* value <.05. GraphPad Prism 7.03, USA was used as software.

## 3. Results and discussion

### 3.1. Construction of pseudoternary phase diagram

**Figure 1.**
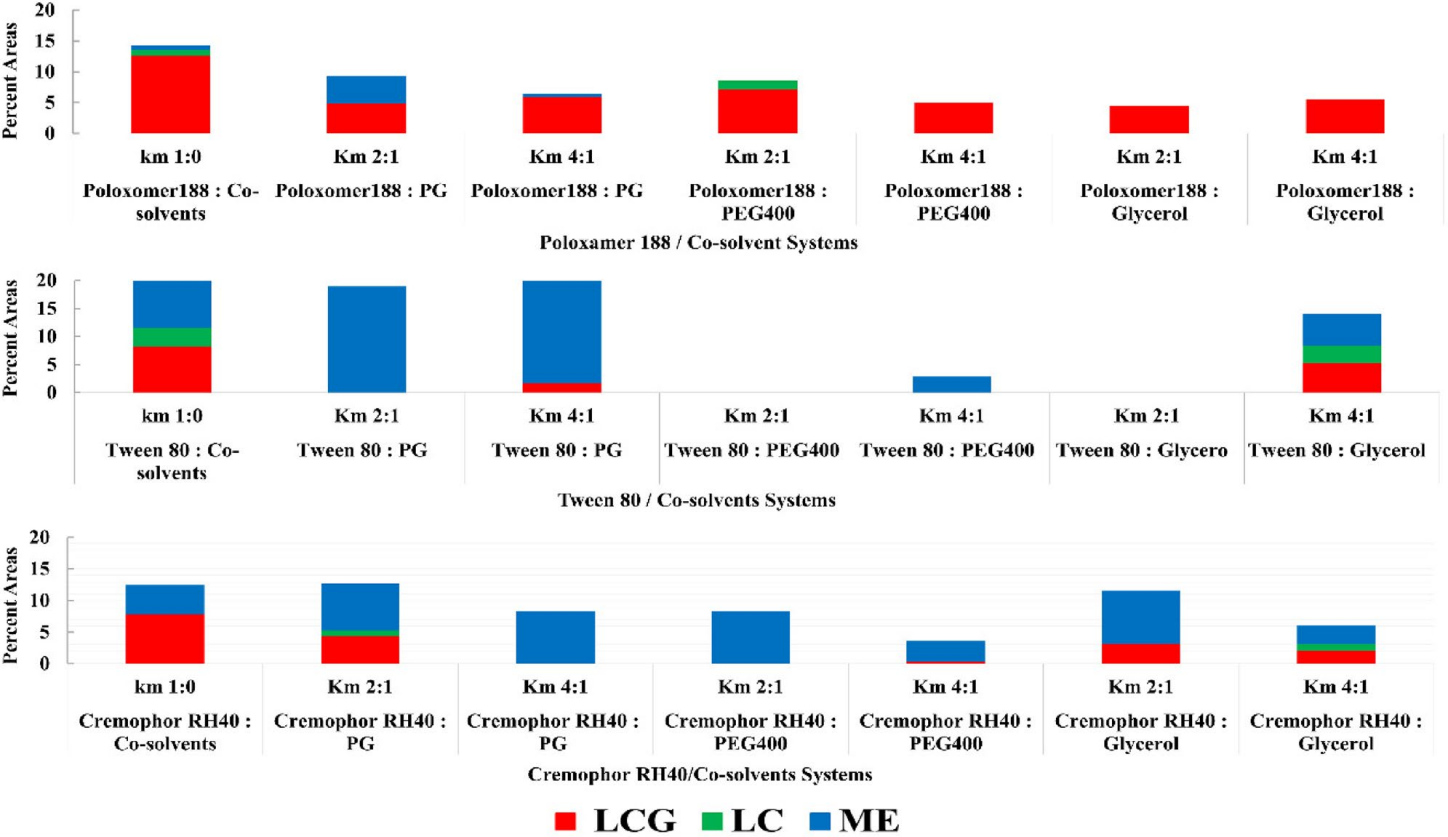
Effect of Km ratios and different systems components on the percent area of ME, LC and LCG.

**Figure 2.**
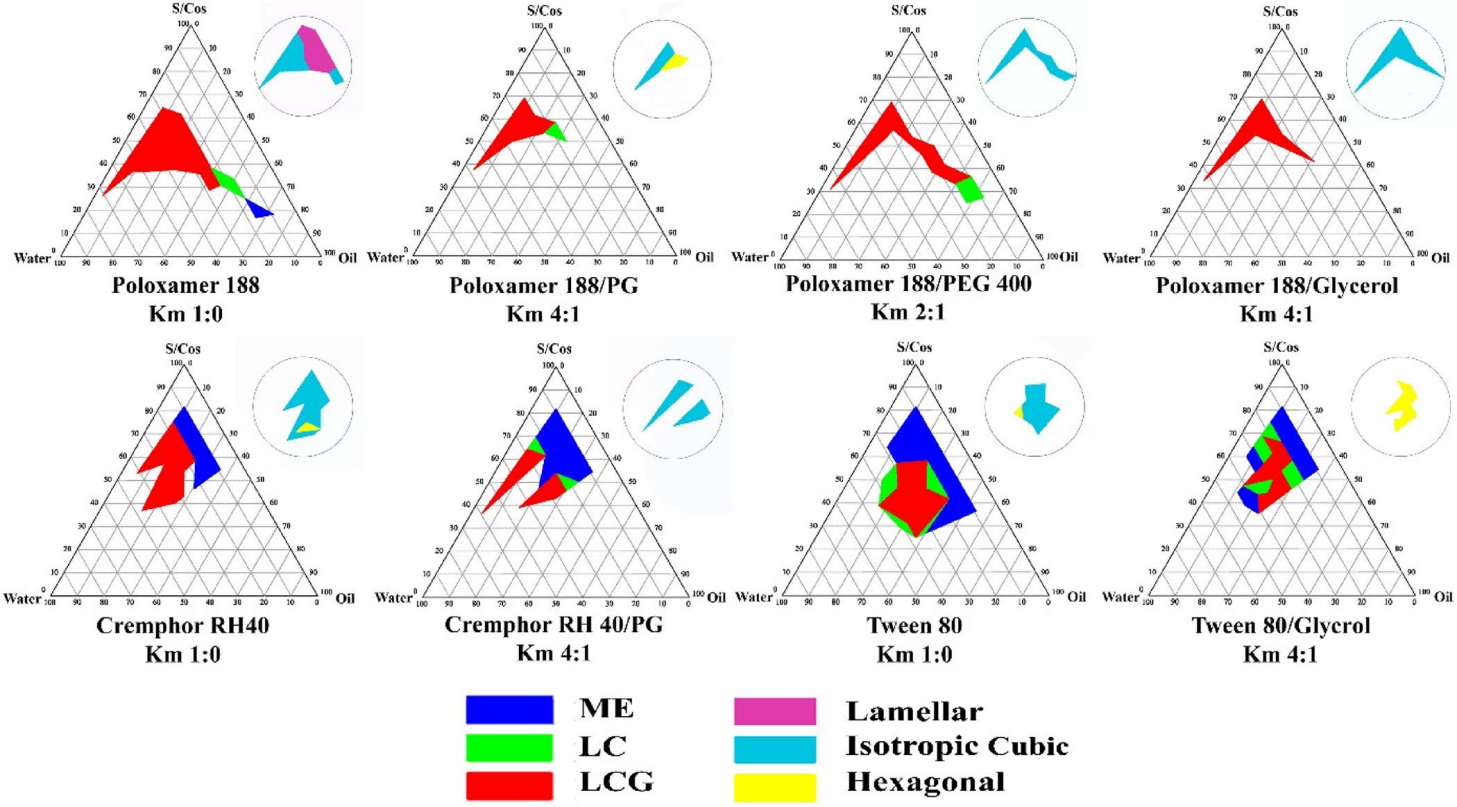
Pseudoternary phase diagram of selected systems for drug loading, clear liquid area denoted ME, clear highly viscous areas denoted LC and clear gel area denoted LCG. Upper corner circles represent the mesomorphic nanostructures of the obtained LCG areas.

The mixture of oil, water, surfactants and co-solvents has the ability to form multiple structures and phases. Structural examination revealed the existence of coarse emulsion, microemulsion and liquid crystalline mesophases. PTPDs were constructed according to visual inspection for clarity and consistency. Effect of *K*_m_ ratios and different systems components on the percent area of microemulsion (ME), LC and LCG are presented in Figure 1. Systems that are able to form large LCG area were further characterized by PLM for identification of the type of the formed mesophase (Figure 2).

The amphiphilic dimensions of the formed mesomorphic structures and their arrangements depend to a large extent on the shape factor of the used amphiphiles ‘critical packing parameter (CPP)’. It is a qualitative theoretical parameter used as rule of thumb and a powerful tool to predict and understand the possible self-assembled aggregates. Such factor is governed by the amphiphilic volume of the hydrophobic chain (*V*), the area of the polar head group (*a*_0_) and the length of the alkyl chain (*l*_c_) according to the following equation (Israelachvili et al., 1976):

(4) $$\text{CPP}=\;V/a_{0}.l_{c}$$

For CPP =1, a planar architecture with zero interfacial curvature is preferred and a lamellar phase is produced, while for CPP < 1 an oil in water mesophase is produced (type I) with a positive interfacial curvature, whereas an inverse water in oil mesophase (type II) is produced with negative interfacial curvature if CPP > 1 (Israelachvili et al., 1976).

In all studied systems, the region of the area occupied by the formed liquid crystals in the phase diagrams complied with the position of the area of inverse type liquid crystals in the generic phase diagram according to Kahlweit & Strey (1985) and Callender et al. (2017).

The obtained inverse phase type LC is consistent with the widely reported inverse phase behavior observed by MO in LC formulation (Tran et al., 2015). Such behavior was engineered through molecular designing concerns that intended to exaggerate the hydrophobic skeleton with respect to the head group. The hydrophobic tail is kinked by insertion of unsaturation in the hydrophobic chain resulting in increasing CPP. In addition, according to Caffrey et al., the inclusion of *cis* double bond in the MO hydrocarbon chain at the 9,10 position maximizes the chain splay (Misquitta & Caffrey, 2001; Fong et al., 2012).

#### 3.1.1. Effect of type of surfactant on the formation of the nanostructured mesophases

Observed data revealed that the tri-block copolymer Poloxamer 188 produced the largest percentage of LCG area (12.6%) followed by Tween 80 (8.3%) then Cremophor RH 40 (7.9%).

Ternary systems composed of MO ‘a cubic phase forming lipid’, with an amphiphile in presence of water can considerably increase the prospects to tune the nanostructure and bilayer properties. In the view of CPP, these observations can be explained by enterprising the complementary shapes of the MO (wedge; type 2 micelles) and the added amphiphilic molecules (Kulkarni et al., 2011; van‘t Hag et al., 2017).

##### 3.1.1.1. System prepared using Poloxamer 188

Investigation of the formed LCGs under PLM revealed the presence of anisotropic structures characteristic of lamellar phase at all (surfactant:oil) ratios and at low water content up to 37.5%. These anisotropic structures converted to isotropic stiff gel at higher water concentration indicating formation of cubic mesophase.

Considering MO, a lamellar phase is reported in MO systems at low water content which is converted to cubic phase as the amount of water increases (Shah et al., 2001). Moreover, it was identified by Tomas Landh that there is a competition between MO and Poloxomer molecules for self-assembly in the bilayer. Such competition is won by the assembly of the MO and the type II cubic phases works as an ‘ideal solvent for the triblock copolymer’. Taking into account the effect of poloxamer on the complementary shape of MO molecule, the observed lamellar phase is consistent with what is proposed earlier; polypropylene segment of the tri-block co-polymer has a favored position just below the interfacial lipid bilayer, causing an increase in the area of the head group and reduction in the negative curvature which reflected in the formation of lamellar phase rather than the cubic one (Landh, 1994). On the other hand, the formation of cubic phase by increasing water content in this system could be attributed to the increased hydrocarbon chain disorder which arises from increasing either the water content or the temperature (Shah et al., 2001). In addition, the higher hydration of the stern layer in the head group diminishes the amount of the non-polar interactions between hydrophobic tails in the core of the formed micelles (Bakshi, 2016).

##### 3.1.1.2. System prepared using Tween 80

In this system, it was figured out that by increasing water content, LC and LCG phases were formed at certain surfactant: oil ratios and up to 44.4% water content. At lower water concentration, the amount of water is not sufficient to hydrate the surfactant polyoxyethylene head groups, such hydration is essential for swelling the amphiphilic molecules to display the gel structure. On the other hand, too much water content will lead to destabilization of the gel structure as a result of the increased distance between the amphiphilic head groups (Syed & Peh, 2014). In terms of surfactant to oil ratio, at higher ratios, isotropic LCG phase was formed which was converted to hexagonal phase as the ratio of surfactant to MO was decreased (Figure 1). Tween 80 has a CPP < 1 due to its large head group, this amphiphilic structure when in combination with MO, encourages transition toward the less curved mesophases. Tween 80 is reported to induce the formation of the less negatively curved sponge phase L3, characterized by larger water channels and fluidity than the inverse cubic phases. The observed optical isotropy and low consistency of this formula support its self-assembly in the sponge mesophase (Chen et al., 2015; Tan et al., 2019).

The formation of the hexagonal phase as the concentration of MO increased relative to the surfactant concentration and at higher water content could be rationalized; as the MO wedge shape factor is relatively more predominant over the cone shape of tween 80. Consequently, the complementary CPP of the two molecules at these ratios is tuned to a larger value than at the lower MO ratios, resulting in the formation of the more negatively curved hexagonal phase. Such effect is augmented by the increased solvent concentration as previously explained by Shah et al. (2001).

##### 3.1.1.3. System prepared using Cremophor RH-40

Mesophases formed by this system were relatively similar to those observed in system containing Tween 80. PLM examination revealed the absence of anisotropic structures at most of the concentration ranges that could give LCG, indicating formation of isotropic cubic phase. By decreasing the percent of Cremophor RH40 to MO, a small area of hexagonal phase was formed at the region of higher water concentration. Cremophor RH-40 is reported to have truncated cone surfactant geometry attained by its inclusion of multiple alkyl chains; such shape factor of CPP >1 prefers a more negative interfacial curvature (Yhirayha et al., 2014). Regarding the molecular geometry of the complementary shapes of MO with Cremophor RH40; formation of inverse non-lamellar phases is favored as a result of the increased CPP (Fong et al., 2012). The same explanation for the formation of inverse hexagonal phase at the lower surfactant to oil ratios in case of Tween 80 could be applied for Cremophor RH 40 system. At these ratios, the contribution of MO wedge shape with its maximum hydrophobic outline is more than the contribution attained by truncated cone shape of Cremophor RH 40. Consequently, the complementary CPP of the two molecules at these ratios is larger than that at the higher surfactant to oil ratios.

#### 3.1.2. Effect of different types of co-solvents on the formed nanostructured mesophases

In the view of practical and industrial applications, investigation of the effect of different commonly used co-solvents in studying drug formulation is of fundamental concerns since they offer a less polar environment for drug molecules and other hydrophobic additives which aid in their solubilization, hence facilitates the upscaling of the target formulation into the industrial application. Each co-solvent exerts its specific complex effect on the formation of LCG which discloses the existence of optimum co-solvent concentration for reaching the maximum LCG phase. Broadly speaking, co-solvents exert their action by altering the CPP by either incorporation into the interfacial film, therefore imparting it a greater fluidity or by reducing the aqueous phase hydrophilicity (Aboofazeli et al., 1994). On the other hand, such co-solvents effect on phase behavior was reflected in the feasibility of the formulation process. It was found that the processability of the LLC systems was facilitated to a great extent by the presence of the two co-solvents PG and PEG 400 while it was remarkably harder in the presence of glycerol.

##### 3.1.2.1. Effect of PG on the obtained systems

The effect of PG on both systems containing Tween 80 and Cremophor RH-40 was similar. At the higher *K*_m_ ratio (4:1), the LCG area substantially shrank; the amount of shrinkage in the percentage of area was from 8.3% to 1.7% and from 7.9% to 4.4% for systems of Tween 80 and Cremophor RH 40, respectively. At the lower *K*_m_ ratio (2:1), LCG area disappeared completely and only microemulsion phase could be obtained. This observation could be attributed to the effect of co-solvent on lowering the interfacial tension and fluidizing the rigidity of the surfactant film, thereby increasing entropy of the system (Lawrence & Rees, 2000).

In terms of CPP, PG may interact with the polar moiety of the amphiphile, resulting in increasing the cross-sectional area of the amphiphilic head group. This reduction in CPP favors the formation of a more spherical shape rather than the planar lamellar structure. This is in accordance to the findings of Yhirayha and his research group studying the effect of the different formulation components on the formation and the nanostructure of the obtained LCs (Yhirayha et al., 2014).

For Cremophor RH40 system, the reduced LCG area showed a preservation of the isotropic mesophase type upon the introduction of PG at *K*_m_ 4:1. For the most hydrophilic solvents, seemingly there is a reduced impetus toward phase transition as these solvents could be lodged within the water channels (van‘t Hag et al., 2017).

In case of Poloxamer 188 system, formation of LCG is preserved at the two examined Km ratios, the values of shrinkage in the % area were close to each other at both ratios (change in % area from 12.6% to 5.9% and 4.9% for *K*_m_ ratios of 4:1 and 2:1, respectively). PLM examination revealed the elimination of the lamellar phase and the formation of isotropic phase with the appearance of hexagonal phase at the area with higher MO concentration.

These observations could be explained in the view of Ivanova et al. who confirmed their developed hypothesis for the role of co-solvents on the phase behavior of binary systems of poloxamer and water according to co-solvents relative polarity. They classified the co-solvents as PEO-alike (favor localization in the polar PEO) and PPO-alike (favor localization in the non-polar microdomains). Accordingly, PG will be located in the PPO hydrophobic domain, thus increasing preference for negative interfacial curvature and supporting the formation of cubic and hexagonal phase over the lamellar phase (Ivanova et al., 2001).

Formation of hexagonal phase at this system could also be related to the relief of the high packing frustration characterized for hexagonal phase that could be attained by partitioning of the added PG molecules into the apolar domain, hence the void volume resulted from the packing frustration is occupied (Fong et al., 2012).

##### 3.1.2.2. Effect of glycerol on the obtained systems

The effect of glycerol on system containing Tween 80 differed according to the used *K*_m_ ratios. At high *K*_m_ ratio, the LCG phase was maintained with shrinkage on its percent area from 8.3% to 5.3%. In addition, ME area shrank from 10.8% to 5.6%. Moreover, PLM examination revealed phase transition from isotropic to hexagonal phases. At the lower *K*_m_ ratio (2:1), both the ME and LCG phase areas were eliminated. This effect of increasing preference for negative interfacial curvature of the formed LCG was also observed in Poloxamer 188 system. As the lamellar phase observed in this system at the region of low water content was eliminated by addition of glycerol and only isotropic phase was obtained at both *K*_m_ ratios, shrinkage in the LCG area from 12.6% to 4.4% and 5.5% at *K*_m_ of 2:1 and 4:1, respectively was observed. These findings could be explained on the basis of glycerol kosmotropic effect (order-making). Kosmotropes incline to generate a competitive microenvironment with the surfactant hydrophilic moiety for the water of hydration. The intramolecular hydrogen bonding between the amphiphilic polar head may increase as a consequence to hydrogen bond formation between the water molecules and kosmotropes which leads to an augmented flexing of the amphiphilic interfacial layer toward the direction of the aqueous domain resulting in increasing the negative curvature and decreasing lattice parameter. Consequently, glycerol is reported to be excluded from the interfacial bilayer and works on stabilization of the bulk water which leads to an increase in the wedge shape of the amphiphilic molecule as a result of the reduction of the area of the head group (Abe & Takahashi, 2003; Kulkarni et al., 2011; van‘t Hag et al., 2017; Tan et al., 2019). Ivanova et al. have also reported that glycerol is positioned only in the hydrophilic domains away from the interfacial bilayer and has no contribution to the swelling of the PEO moieties of the block copolymer (Ivanova et al., 2002).

The effect of glycerol on Cremophor RH 40 system indicated that at the higher *K*_m_ ratio (*K*_m_ 4:1), both LCG and microemulsion areas shrank considerably (7.9% to 2.1% for LCG and from 4.5% to 2.9% for ME). At the lower *K*_m_ ratio (2:1), only the LCG area shrank from 7.9% to 3.1%, while the ME increased from 4.5% to 8.4%. This expansion on the ME area may be attributed to the fluidizing effect on the interfacial film of the surfactant imparted by glycerol as co-solvent while the glycerol content at higher ratio was not abundant to impart this action.

##### 3.1.2.3. Effect of PEG 400 on the obtained systems

Considering LCG area, PEG 400 eliminated the LC area in both Tween 80 and Cremophor RH40 systems at the two investigated *K*_m_ ratios. Considering microemulsion formation, ME area highly shrank at the higher *K*_m_ ratio, and nearly disappeared at the lower *K*_m_ ratio in system containing Tween 80, whereas in Cremophor RH40 system, the reduction in ME area was less drastically compared to the Tween 80 system as it shrank from 4.5% to 3.2% at *K*_m_ 4:1, while it was expanded from 4.5% to 8.3% at the lower *K*_m_ 2:1 as shown in Figure 1. Elnokaly et al. realized that the increase in the length of the chain of alkane diol molecule decreases the stability of the structure of lamellar LC, long chain alkane diols were mainly localized between the surfactant molecules at the interface, while shorter chain alkane diol were localized primarily in the aqueous domain between the surfactant layers, positioning the co-solvent molecules at the interfacial film region leading to destruction of the LC phase (El-nokaly et al., 1981; Aboofazeli et al., 1994). These findings could also be explained according to Leonie van‘t Hag et al. who reviewed the effect of high-molecular weight molecule in the formation of nanostructured phases, namely, dextran and PEG. They stated that phase separation occurred when the hydrodynamic diameter of the added soluble molecules exceeds the diameter of the aqueous channels of the nanostructured formed phase (van‘t Hag et al., 2017).

The effect of both PEG 400 and glycerol on the formation of ME or LCG mesophase was more drastic in the case of Tween 80 rather than Cremophor RH 40, that may be explained in the view of the number of monoethylene oxide moieties in the two molecules. Cremophore RH 40 contains double the number of polyethylene moieties in the head group, and three times the number of hydrophobic chain, hence the interaction between the two surfactant moieties and the co-solvent molecules may need larger amounts of co-solvent molecules to exert the same effect observed in the case of the system of Tween 80.

The preservation of the LCG area by using PEG 400 was observed only in Poloxamer 188 system, the LCG area shrank from 12.6% to 5% and 7.2% at *K*_m_ 4:1 and 2:1, respectively. PLM examination revealed the formation of isotropic phase only indicating preference of increasing negative interfacial curvature. According to Ivanova et al., PEG 400 belongs to the ‘PEO-resembling solvents’, its effect on phase behavior is the same to that of glycerol. As a result, PEG 400 positioned only in the polar domains and has no contribution to the swelling of the PEO blocks. Both PEG 400 and glycerol are favorably solvated by water molecules and hence results in de-swelling of the PEO blocks (Ivanova et al., 2002).

Regarding the LCG percent area and the compatibility with all types of the used co-solvents, the previous results disclose the superiority of the tri-block copolymer over the conventional surfactants for the formation of LCG phase over a wider range of components concentrations. In systems of amphiphilic block copolymers, the arrangement of chemically different blocks in a flexible long polymer chain results in unmatched properties in both versatility in self-assembly and tunability by other additive molecules in nanostructures formulations (Bodratti & Alexandridis, 2018; Reddy et al., 2021). The excessive amount of hydration of the polymeric monomers, the longer hydrophobic chain results in a highly condensed interfacial layer because of the more powerful interaction between the hydrophobic tails. On the other hand, in systems of conventional amphiphiles, the diversity of the formed nanostructures is restricted by the simple geometrical constraints which fix the preferred curvature (Ivanova et al., 2001).

### 3.2. Preparation of CFZ-loaded NLCG formulae

Preliminary formulation of different drug-loaded concentrations revealed that 4% drug concentration was the maximum concentration that could be loaded in the chosen formulae without affecting its physical characters: namely, clarity and consistency. Maximum drug concentration was used to maximize the concentration gradient driving force required for skin permeation process. Compositions of each 4% drug-loaded NLCG formulae are shown in Table 1.

**Table 1.** Composition of 4% Canagliflozin loaded NLCG formulae.

Drug-loaded formula	% Composition			
	Oil (MO)	Amphiphile	Co-solvents	Water
NLCG 1	6.5	57.5 (Poloxamer 188)	–	32
NLCG 2	6.5	46 (Poloxamer 188)	11.5 (PG)	32
NLCG 3	6.5	38 (Poloxamer 188)	19 (PEG 400)	32
NLCG 4	6.5	46 (Poloxamer 188)	11.5 (Glycerol)	32
NLCG 5	6	54 (Cremophor RH 40)	–	36
NLCG 6	6	43 (Cremophor RH 40)	11 (PG)	36
NLCG 7	22	52 (Tween 80)	–	22
NLCG 8	22	52 (Tween 80)	9 (Glycerol)	22

### 3.3. Characterization of CFZ loaded NLCG formulae

#### 3.3.1. Morphological examination

Polarized light microscopy is considered the standard and simplest used technique for characterization and identification of the basic phase behavior of the bulk viscous gel phases. PLM requires no special treatment for sample preparation and hence no phase change or deformation could occur. Moreover, in the occasion of optically active birefringents, such as anisotropic liquid crystals, upon entrance of light, its ray is split by polarization into two, the fast known as the ordinary ray and the slow called the extraordinary ray, taking slightly different paths. Because the two components travel at different velocities, the waves get out of phase. When the rays are recombined as they exit the birefringent material, the polarization state has changed because of this phase difference. Thus, investigations of birefringent materials are mostly performed with polarized light, as it strongly interacts with the sample generating contrast with the background, allowing focusing on details not visible with unpolarized light (Voelker-Pop, 2014). On the contrary, in electron microscopic characterization where the bulk viscous LLC sample is not amenable to the approaches of sample preparation such as vacuum drying and cooling techniques. Such approaches represent a limitation in using electron microscope for characterization of the bulk LLC.

The small-angle X-ray diffraction could be used for further characterization of the lattice parameters, however, the data is difficult to interpret for complex or heterogeneous phases. Yet, such subtle characterization is beyond the aim of this study (Garti et al., 2012; Milak & Zimmer, 2015).

**Figure 3.**
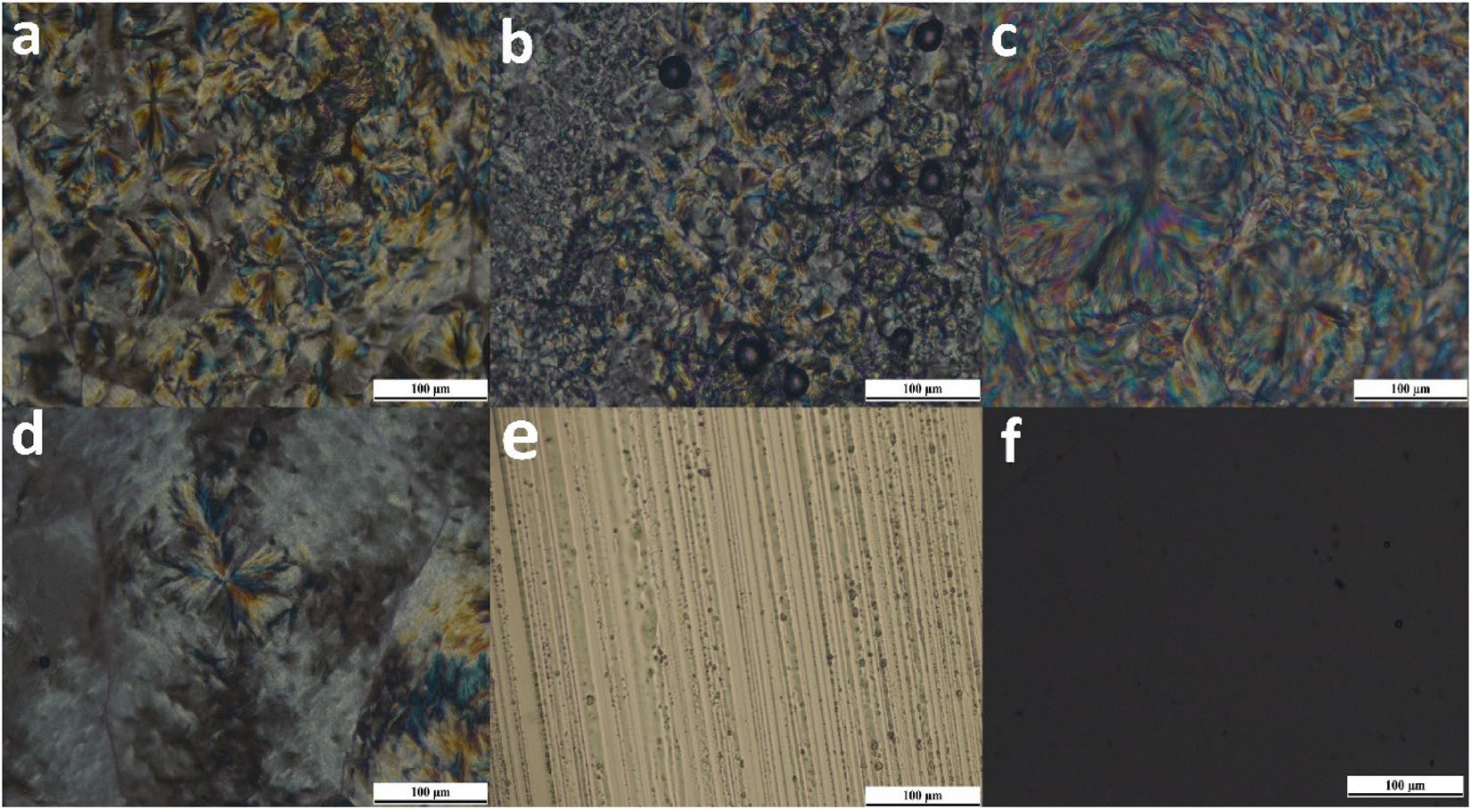
Different microscopic textures as shown by PLM: (a–d) characteristic lamellar phase maltese crosses, (e) characteristic hexagonal non geometric periodic texture, (f) dark field indicating isotropic phase.

The observed results for PLM investigated samples of the drug-loaded formulae are shown in Table 2. The observed characteristic textures of each phase are shown in Figure 3.

**Table 2.** Results of physical characterization of CFZ NLCG formulae.

Drug loaded formula (% composition)	Visual inspection (Consistency-Clarity)	Viscosity at min. SR (cP)	Type of mesophase	pH
NLCG 1	Clear stiff gel	11,430 ± 571	Lamellar	4.5
NLCG 2	Clear stiff gel	15,637 ± 783	Isotropic	4.3
NLCG 3	Clear stiff gel	17,145 ± 857	Isotropic	4.4
NLCG 4	Clear stiff gel	15,478 ± 773	Isotropic	4.4
NLCG 5	Clear gel	14,367 ± 718	Isotropic	5.3
NLCG 6	Clear soft gel	4,604 ± 230	Isotropic	5
NLCG 7	Clear gel	2,540 ± 127	Isotropic	5.5
NLCG 8	Clear gel	15,637 ± 781	Hexagonal	5.5

Isotropic cubic liquid crystals in contrast to the anisotropic liquid crystals (lamellar and hexagonal), do not interfere with the polarized light, the field of view remains dark as there is no deviation of the light and the polarizer absorbs the light passing through it (Figure 3(f)) (Djekic & Primorac, 2008).

Hexagonal and lamellar anisotropic mesophases cause a deviation in the plane of polarized light, resulting in a birefringent character that displayed as a black and white image, or colored textures. The lamellar phase showed characteristic Maltese crosses shape results from concentric rearrangement of plane layers and are the dominant texture with oily stripes (Figure 3(a–d)) (Silvestrini et al., 2020). Hexagonal characteristic non-geometric periodic texture that related to column undulations was also observed (Figure 3(e)) (Livolant & Bouligand, 1986; Mourad et al., 2010; Dutt et al., 2017; Raspantini et al., 2021).

#### 3.3.2. Evaluation of the pH of the selected formulae

The optimum pH value for good skin condition is reported to be in the range of 4–6. pH values of the chosen formulae are shown in Table 2. All values fall within the acidic range that is compatible for good skin condition, especially poloxamer formulae which fall in the optimum value of the required pH. Acidic pH is important for stratum corneum (SC) homeostasis, also for keeping optimum structure necessary for maintaining skin lipid barrier properties. Long-term treatment of skin with formulae of acidic pH values that ranged between 3.5 and 4.0 could normalize the elevated values of skin pH in the geriatric population leading to improved skin barrier integrity and imparting an enhancement in skin condition (Blaak et al., 2011).

#### 3.3.3. *In vitro* permeation study

Two dose protocols are applied in modeling *in vitro* transdermal drug delivery; namely, infinite and finite dose. In infinite dosing regimen, the applied dose is in large excess that depletion of the permeant in the donor compartment is negligible, therefore the dose is considered to be constant and infinite. On the other hand, in finite dose regimen, only a limited amount of the donor formulation is used. Thus the applied dose and exposure time are best related to the *in vivo* situation and prospective human exposure (Lau & Ng, 2017). For semisolid and solid substances, values ranged from 1 to 10 mg/cm^2^ are considered as finite dose condition (Selzer et al., 2013). Unfortunately, mathematical expressions for treatment of data from finite dose experimental regimen are extremely complex and are not feasible to routine use, consequently, there is no simple method for the calculation of the permeability coefficient *K*_p_ from finite dose experiments (Mitragotri et al., 2011; Lehman, 2014). The maximum flux (*J*_max_), the time to maximum flux (*T*_max_) and the total amount of drug permeated in a given time through a given area (*Q*) are the most commonly reported parameters in finite dosing protocol (Finnin et al., 2012; Lau & Ng, 2017).

The use of animal skin could give misleading results because of the existed dissimilarity to the human skin which imparts imperfection in extrapolating the data obtained from animal species to that obtained in humans. Consequently, utilization of artificial and simulated skin attracted researchers due to ease of preparation, uniformity in composition and reproducibility in the obtained results (Neupane et al., 2020). Eggshell membrane mimics human SC (the rate limiting step in skin permeation), consists mainly of a meshwork of interwoven fibrous structure of keratin and 3.5%–4% lipid. The main lipid components are glycerides and cholesterols, free fatty acids in addition to an amount of phospholipid represented as lecithin, cephalin and sphingomyelin (Hamilton, 1986; Ansari et al., 2006).

**Figure 4.**
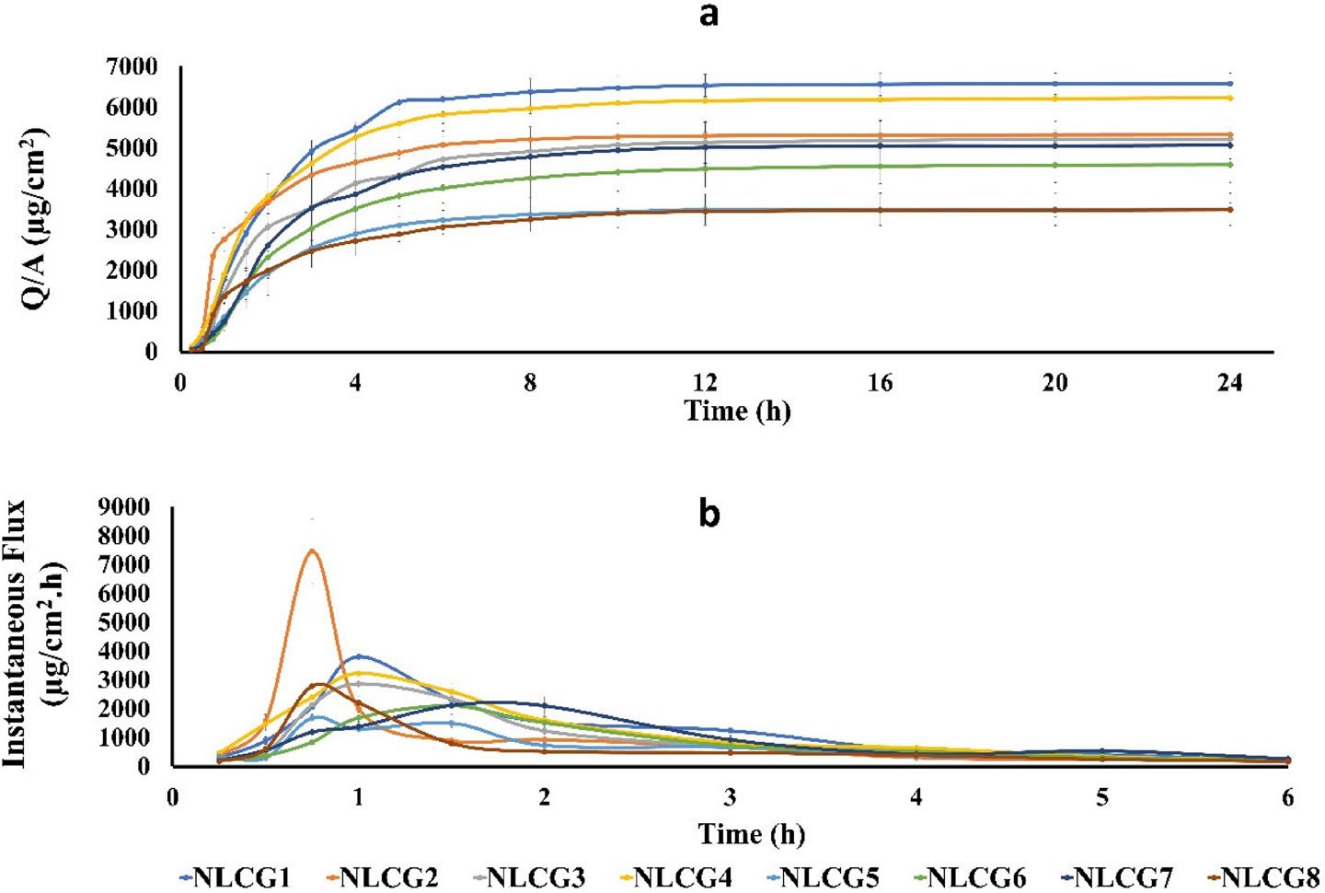
**(**a) Canagliflozin cumulative amount permeated per unit area from the selected formulae and (b) Canagliflozin maximum flux (*J*_max_) according to amount permeated per time (instantaneous flux).

Results of permeation study are shown in Table 3 and Figure 4. Considering the three permeation parameters (*J*_max_, *Q*_24_ and *T*_max_), the results revealed that formulations containing Poloxamer 188 as a surfactant (NLCG 1, 2, 3 and 4) showed the highest parameters of *J*_max_, *Q*_24_ and *T*_max_, respectively, among the tested formulae. The other formulae showed comparably lower permeation parameters.

**Table 3.** Permeation parameters of canagliflozin NLCG formulae.

Formula	Maximum flux *J*_max_ (µg/cm^2^ h)	*T*_max_ (h)	Cumulative amount permeated (*Q*_24_) (µg/cm^2^)
NLCG1	3,805 ± 559	1	6,582 ± 259
NLCG2	7,460 ± 1,117	0.75	5,327 ± 336
NLCG3	2,867 ± 416	1	5,215 ± 482
NLCG4	3,225 ± 318	1	6,224 ± 43
NLCG5	1,680 ± 226	0.75	3,495 ± 110
NLCG6	2,101 ± 292	1.5	4,588 ± 432
NLCG7	2,114 ± 283	1.5	5,067 ± 397
NLCG8	2,788 ± 60	0.75	3,486 ± 163

These findings can be explained mainly in the view of the type of the microstructure of the formulated LLC which is depicted according to both PLM phase texture and consistency of the formulated NLCGs (Phan et al., 2011; Streck et al., 2016). For formulae NLCG2, 3 and 4, the stiff gel consistency and its isotropic properties revealed their existence in the bi-continuous cubic phase while NLCG1 exhibited lamellar phase texture. Cubic phase gel enhances transdermal permeability by virtue of the state of the water channels which is opened in the case of cubic phase (Lv et al., 2005; Chen et al., 2007; Phan et al., 2011). In addition, the unique three dimensional structure of periodically curved lipid bilayer and the nano-aqueous channels network with a tremendous estimated value of interfacial surface area of 400 m^2^/g in the cubic mesophases impart faster drug transfer and diffusion and afford the main path for both hydrophilic and lipophilic molecules to release (Boyd et al., 2006; Guo et al., 2010) .

The highest maximum flux (*J*_max_) value of 7460 μg/cm^2^ h was obtained by formula NLCG 2 which shows a marked significant difference compared to all other formulae at *p <* .0001. The slower permeation rate of the other poloxamer cubic gel formulae (NLCG 3 and 4), may be related to the almost narrower water channels in these formulations as a result of the de-swelling of the amphiphilic head group caused by glycerol and PEG 400 that gave rise to a more negatively curved cubic phase with a reduced lattice parameters (Chen et al., 2014).

On the other hand, the highest *Q*_24_ value (6582 μg/cm^2^) was shown by the lamellar NLCG1 (significantly different compared to all other formulae (*p <* .05) except NLCG4). Such high *Q*_24_ value could be due to conversion of the lamellar phase to the cubic phase as water content increases during membrane permeation. This explanation has been studied and confirmed by Eder Andre Estracanholli et al. and Dae Gon Lim et al. who submitted their systems to phase identification by PLM after the drug release studies (Estracanholli et al., 2014; Lim et al., 2014). Lacking of co-solvent in NLCG1 may have resulted in the formation of larger water channels compared to the other poloxamer formulae and thus could be responsible for its higher *Q*_24_ value.

Permeation parameters of the other amphiphilic systems of Tween 80 and Cremophor RH40 have shown significantly reduced values compared to that of Poloxamer 188 group. The smallest *Q*_24_ values (3495, 4588, 3486 μg/cm^2^) have been showed by formulae NLCG 5, 6 and 8 respectively. No significant difference (*p >* .05) was found between the three formulae. In addition, no significant difference was found in the *J*_max_ values (1680, 2101, 2114 and 2788 µg/cm^2^ h for NLCG 5, 6, 7 and 8, respectively). Cremophor RH40 containing formulae (NLCG 5 and 6) exhibited isotropic properties under PLM and their consistency was far from being stiff gel suggesting their existence in the less viscous micellar form cubic gel. For sustaining drug release, micellar phase – which is composed of closed structure of two different sizes of discrete micelles embedded in a continuous three-dimensional hydrophobic matrix is presently one of the most widely studied phases for that purpose (Fong et al., 2012; Huang & Gui, 2018). Phan et al. reported much more slower release rate by GMO-based micellar phase type in comparison to lamellar phase despite its lower viscosity (Phan et al., 2011). Retarded release rate of doxorubicin in micellar poloxamer gel formulations was also reported and the results were attributed to the hindrance of water mobility inside the gel (Bodratti & Alexandridis, 2018). The low *Q*_24_ value of NLCG 8 could be related to its existence in the hexagonal mesophase type. In the reversed hexagonal phase, the aqueous domain is a highly ordered structure of closed water channels thus it is responsible for the slow drug release and consequently low permeation parameters (Boyd et al., 2006; Phelps et al., 2011; Garti et al., 2012; Huang & Gui, 2018). Vallooran et al. have reported another interesting explanation that may be accountable for the slow drug diffusion from hexagonal phase which is the random alignment of the domains of the bulk hexagonal mesophase that diminishes the diffusion of the drug molecules. This non-uniform orientation leads to a significant reduction in drug diffusion resulting from the interrupted pathway that faced drug molecules. Accordingly, in anisotropic phases, the rate of diffusion is controlled by such domain orientation as well as the dimensions and state of the water channels (Vallooran et al., 2013). On the other hand, in spite of the low permeation values showed by NLCG 5 and 8, they displayed low value of *T*_max_ (0.75 h) which may be attributed to an initial rapid release of a portion of the drug located at the border of the outer regions of the bilayer that constituting the hydrophobic domain of the micellar cubic and hexagonal phases, followed by slow release of the remaining portion of the drug embedded in the inner layers.

Formula NLCG 7 showed an intermediate permeation behavior in terms of the total amount of drug permeated (*Q*_24_), as previously discussed, NLCG 7 was concluded to exhibit sponge mesophasic type. Sponge phase is considered as a disordered bi-continuous cubic phase characterized by infinite periodic minimal surface (IPMS) of the same topology of that of the cubic phase but with larger water channels which could demonstrate the enhanced permeation that represented in the high *Q*_24_ value of 5067 µg/cm^2^ (Silvestrini et al., 2020, Lim et al., 2014, Ridell, 2003).

#### 3.3.4. Rheological study

With regards to the value of viscosity at minimum shear rate (SR), all formulae showed high viscosity values which are feature of the liquid crystal state of matter (Table 2). Highest values were shown by the cubic and hexagonal formulae (NLCG 2, 3, 4, 5 and 8). A significantly lower value was shown by the lamellar formula NLCG 1 (*p <* .0001). While the significantly different lowest values (*p <* .0001) have been shown by the micellar cubic NLCG6 and the sponge phase formula NLCG7 with the sponge one being the significantly lowest than the micellar (*p <* .003).

Existing opinions reach agreement that the bi-continuous three-dimensional cubic phases are the most firm liquid crystal mesophases, followed by the reversed hexagonal phase and then the lamellar phase which might be described as a plastic fluid (Sagalowicz et al., 2006).

Micellar cubic phase is reported to have a lower viscosity than the hexagonal and the bi-continuous cubic phase (Phan et al., 2011). However, the very low viscosity value showed by the micellar cubic NLCG 6, may be ascribed to the presence of the co-solvent PG which works on reducing the rigidity of the interfacial bilayer of the micellar nanoaggregates.

On the other hand, the extremely lower viscosity value was observed by the sponge phase formula NLCG 7. Sponge phase is a disordered style of the bi-continuous cubic phase with a highly flexible interfacial bilayer and much wider water channels, hence much more fluidity (Imura et al., 2007; Chen et al., 2015).

**Figure 5.**
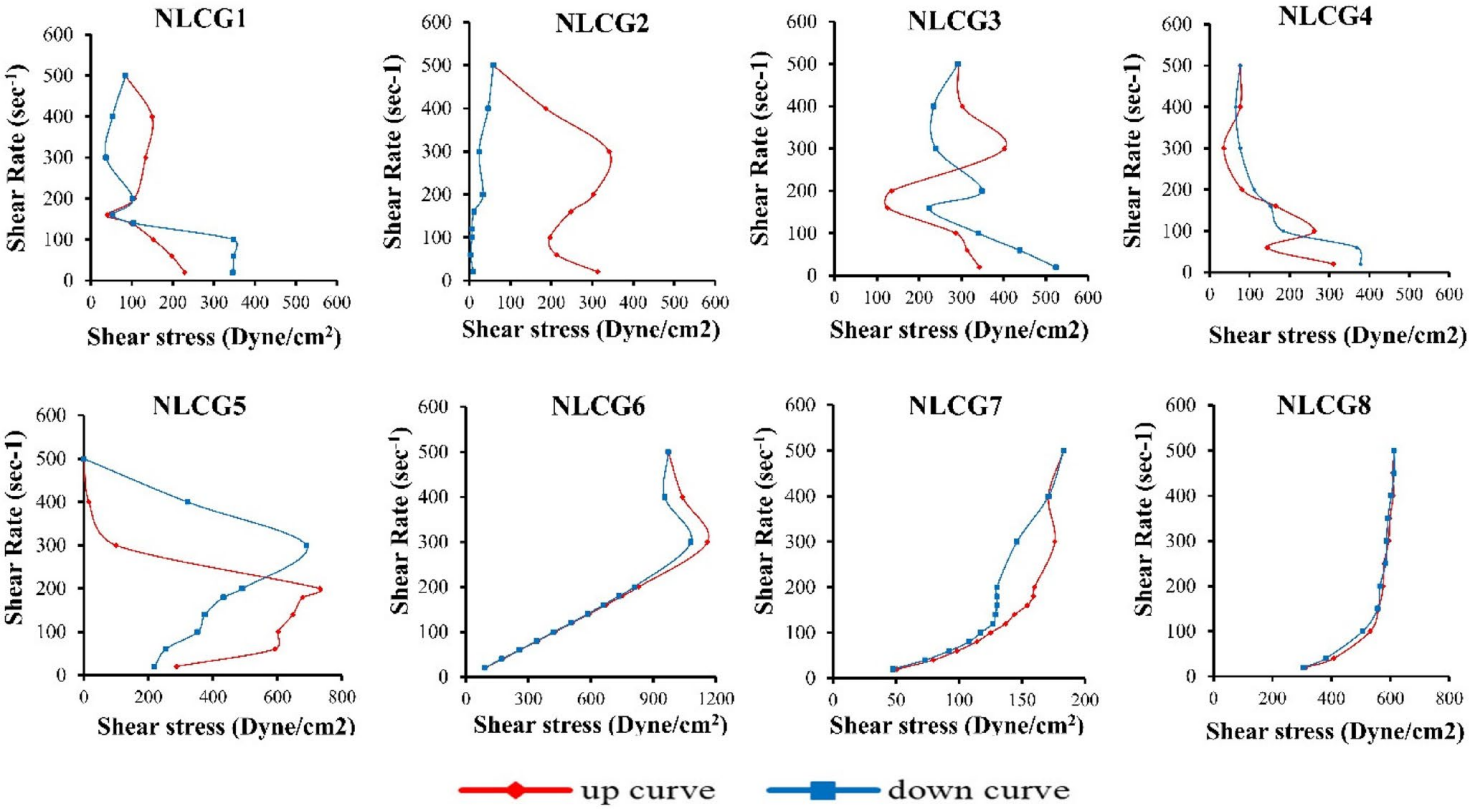
Rheograms of canagliflozin loaded NLCG formulae showing the relation between shear stress and shear rate.

In view of the effect of shear rate on viscosity and shear stress (Figure 5), all formulae generally exhibited shear thinning behavior as the viscosity was decreased by increasing the shear rate, while the effect of shear rate on shear stress was variable and complicated for most formulae; namely, the lamellar NLCG 1and the cubic NLCG 2, NLCG 3, NLCG 4 and NLCG 5. They showed multiple behaviors of shear thickening followed by shear thinning or vice versa along the applied shear rate values. This rheological performance was accompanied by rheopectic and/or thixotropic behavior as well. It has been reported that complex state of matter, such as NLCG, acquired complicated rheological response to the applied shear rate (Soltero et al., 1995). Cubic phases, in particular, are perplexing systems for rheological studies, since their response to stress is a consequence of numerous relaxation mechanisms; a structure transition from phase to another that takes place in response to the applied shear is responsible for this complicated rheological behavior (Mezzenga et al., 2005). The observed rheopexy may be ascribed to formation of shear-induced microstructures at the higher shear rates which results in shear thickening, upon reducing shear rate, much of the formed structure is still preserved and consequently its viscosity remains higher than the values measured in the increasing shear rate mode (Soltero et al., 1995).

Sagalowicz et al. reported the rheological characterization of liquid crystalline mesophases and studied the order–order and order–disorder transformations that occur when shear is applied, they reported cubic to cubic, cubic to hexagonal, and hexagonal to isotropic microstructure transition (Sagalowicz et al., 2006). Similar conclusion was also reported by Berni et al. who studied the rheology of the lamellar liquid crystalline phase. There is a critical stress value for a given constant shear rate, suggesting a microstructural build up until a critical point, at which the initial microstructure can no longer support the deformation and hence must change to a new state (Berni et al., 2002).

Formula NLCG 6 showed Newtonian behavior at shear rate values up to 300 rpm, above such value a shear thickening behavior was observed. In addition, the down-curve showed a higher viscosity at this shear rate than the up-curve indicating a structure transition to another phase that takes place in response to the applied shear. The presence of propylene glycol in the formula softened the formed NLCG, as a co-solvent, it interrupts the assembly of the surfactant molecules in the interfacial surfactant film layer causing a reduction in the film rigidity to a degree that the phase approaches the composition of microemulsion which is characterized by Newtonian flow behavior. However, at the higher shear rate up to 500 rpm, it possessed the highest values of viscosity among all other formulae; it seems that the micelles interact with each other in a way that leads to increasing its resistance to flow, i.e., increase in its viscosity.

Hexagonal formula NLCG 8 showed a plastic flow behavior, as there was an increase in the shear stress values along the whole range of applied shear rates, this shear thinning may be attributed to the initial modest orientation of the amphiphilic rods along the direction of flow followed by a progressive further increase in alignment corresponding to the progressive development of shear thinning. Because of the arrangement of the hexagonally packed aggregates, they can move freely only along their lengthwise direction (Mortensen, 2001).

#### 3.3.5. Accelerated stability study for physical parameters

Ambient storage conditions disclosed physical instability for both Cremophor/PG (NLCG 5) and Tween 80 (NLCG 7) formulae, as a remarkable change in their consistency from gel to viscous liquid was observed. Other formulae that were subjected to accelerated stability at temperature of 40 °C and 75% relative humidity showed good physical stability, except poloxamer 188 formula (NLCG 1), which transformed from transparent to opaque gel indicating phase instability.

#### 3.3.6. Choosing the optimized CFZ-NLCG

Based on the obtained observations and on the results of permeation study, formula NLCG 2, which acquired the significantly highest value of maximum flux, was selected for further evaluation.

##### 3.3.6.1. Chemical stability study of the optimized CFZ-NLCG

The chosen formulation (NLCG 2) was further investigated for its chemical stability. The obtained results of analysis by HPLC revealed high chemical stability for NLCG 2. The average amount of the drug assayed was within the range of 95%–105% of the initial drug concentration, no peaks of drug degradation were observed in the chromatograms.

##### 3.3.6.2. *In vivo* pharmacodynamic evaluation of CFZ-NLCG2 transdermal formula

###### 3.3.6.2.1. Effect of application of CFZ-NLCG2 transdermal formula on blood glucose level in hyperglycemic fasting mice 

The fasting mice test model is a common method performed to reduce variability in the examined parameters. Indeed, a plain transdermal patch was also applied to non-transdermal treated groups to unify the possible factor of stress hyperglycemia in all groups.

**Figure 6.**
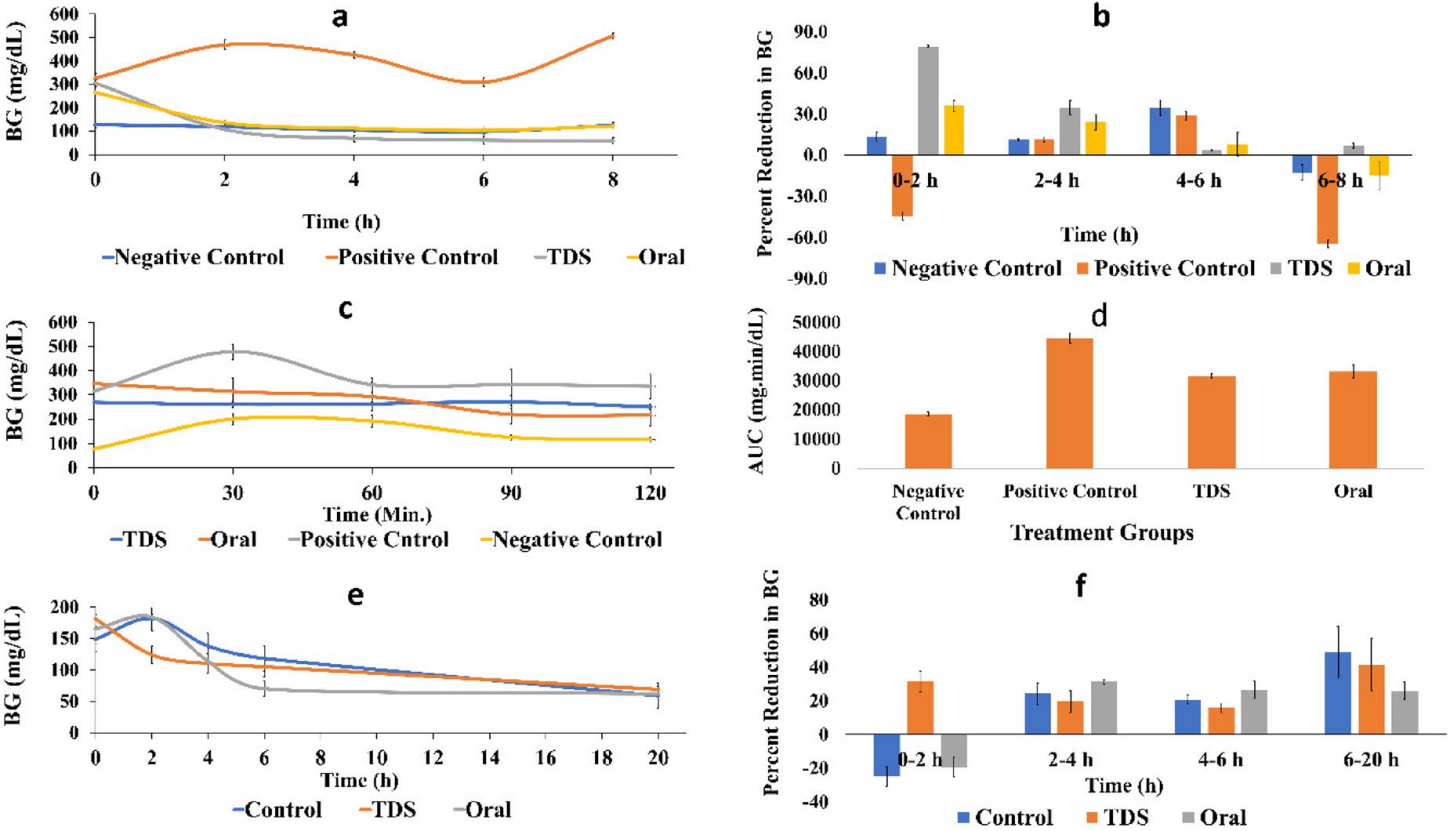
**(**a) Effect of canagliflozin transdermal formula on BG levels in hyperglycemic fasted mice, (b) percent reduction in BG levels in hyperglycemic fasted mice, (c) BG levels in OGTT, (d) BG area under the curve (AUC) in OGTT, (e) effects of canagliflozin transdermal patch on BG levels in normoglycemic mice, and (f) percent reduction in BG levels in normoglycemic mice.

The hyperglycemic effect of STZ was noticed at zero time and sustained till the end of the 8 h with a fluctuating pattern to be always significantly different from the normal healthy mice. After 2 h, the STZ elevated BG to 44% relative to its zero time, and the treatment with either drug formula succeeded to decrease this elevation. However, a significant difference in BG levels (Figure 6(a); *p =* .0145) and the percent reduction in BG level (Figure 6(b); *p =* .02) was detected between the two treated groups after the first two hours. Nevertheless, the hypoglycemic effect of the two treatment regimens was not significantly different at the 4 and 6 h. The transdermal route showed a subtle yet significant decrease in BG relative to that of the oral treated group and the healthy normal ones. Accordingly, our results demonstrate the effectiveness of the transdermal NLCG2 formula that is the same or even overcome that of the per oral route in delivering CFZ to the blood circulation.

This is quite an achievement as loading CFZ on NLCG2 formula succeeded in overcoming the skin barrier to reach the blood circulation at therapeutic levels. Indeed, the potentiated antihyperglycemic activity of the transdermal route allows further reduction in the drug therapeutic dose. Such accomplishment could be attributed to structure and components of the chosen formula. Regarding the structure of the isotropic cubic mesophase, the reported packing symmetry between the SC keratin and the structure of the cubic phase facilitates transdermal delivery through creation of a permeation pathway. This similarity was also suggested to enhance the strong bio-adhesive property to the skin. Such bioadhesive property is specially enhanced in GMO-based cubic phase and thus contributes to a fast permeation rate. Cubic phases are also reported to generate microscopic clustering of the skin keratinocytes creating microfissures pathways (Chen et al., 2014; Silvestrini et al., 2020). Liquid crystal enhancement of transdermal delivery might also be affected by the liquid crystal inter-lamellar water which functions as a reservoir for controlling skin hydration (Rajabalaya et al., 2017).

Monoolein, owing to its hydrophobic head group and its relatively short hydrophobic chain, could enhance transdermal drug permeation by reversibly disrupting the ordered lamellar structure of the SC and increase the fluidity of its lipid (Lim et al., 2014; Silvestrini et al., 2020). In addition, as a lipid, it can loosen the packing of protein by modifying the conformation of keratin causing its swelling and increasing its hydration, or by altering cohesion between corneocytes (Kováčik et al., 2020).

Considering Poloxamer 188, the hydrophilic ethylene oxide moieties in poloxomers are inserted in the polar head group of the lipid membrane by transient interaction via hydrogen bonding. This insertion produces a change in the lipid domain environment that facilitates skin permeation (Bodratti & Alexandridis, 2018; Carrer et al., 2020). The presence of propylene glycol could also play a role in the enhancement of the transdermal delivery. Generally, glycols are able to improve skin permeation by different ways, such as extraction of protein and lipids, SC swelling, enhancing partitioning of the drug into the skin and increasing drug solubility in the formulation (Karande & Mitragotri, 2009). On the other hand, the observed initial fast drug release by CFZ NLCG2 creates an initial high concentration gradient required for efficient transdermal delivery of the drug.

###### 3.3.6.2.2. Effect of CFZ-NLCG2 transdermal formula on OGTT in hyperglycemic mice 

The OGTT is used to address how quickly exogenous glucose can be cleared from the blood. The presented data show an elevated BG that was significantly (*p =* .015) above that of the normal animals as depicted in Figure 6(c). On the other hand, treatment with CFZ in either route inhibited BG levels in the same pattern to reach a significant level (*p* = .0008) from the untreated hyperglycemic mice, however, their effect was still significantly higher than the normoglycemic levels. The same results were mirrored in the AUC values shown in Figure 6(d). Although the transdermal route showed a significant hypoglycemic effect (at 2 and 8 h) as compared to the oral one in the fasting mice model (Figure 6(a,b)), the equal antihyperglycemic activity of the two routes in this test model may be attributed to the inhibitory effect of oral CFZ on intestinal SGLT1 besides its action on the renal SGLT2, where glucose ingestion stimulates the secretion of SGLT1. This notion is confirmed previously, whereas although CFZ selectively inhibits SGLT2 more than SGLT1, yet upon its existence in the intestine, it can also decrease the activity of SGLT1 to delay postprandial intestinal glucose absorption (Faillie, 2017). Moreover, the inhibition of SGLT1 was reported to stimulate the secretion of the incretin hormones; namely, glucose-dependent insulinotropic polypeptide and glucagon-like peptide that are responsible for two- to threefolds increase in insulin secretion in response to oral glucose relative to the intravenously administered glucose (Incretin effect) (Oguma et al., 2015; Nauck & Meier, 2018; Dominguez Rieg & Rieg, 2019). According to the OGTT, our results support the beneficial effect of the transdermal route over the oral one, since the former avoids the GIT side effects mentioned earlier (Tsimihodimos et al., 2018) such as osmotic diarrhea, dehydration and increased bacterial carbohydrate fermentation in the intestine.

###### 3.3.6.2.3. Effect of CFZ NLCG2 transdermal formula on blood glucose level in normoglycemic mice 

The normoglycemic animal model test is used in conjunction with other hyperglycemic diabetic animal models as a valid method for screening the hypoglycemic effect of the drug on healthy animals with intact pancreatic activity.

As depicted in Figure 6(e), a significant decline in BG was noticed after 2 h of food deprivation in the transdermally treated group relative to their basic level, while that of the normal untreated animals and those receiving CFZ by the oral route showed an elevation in BG compared to its zero time. The 2 h differences reached a significant level (*p =* .007 and .004 for untreated and orally treated groups, respectively). This was further reflected on the percent reduction of BG as presented in Figure 6(f) to point for the ability of the transdermal route to level off BG more than either the oral route or the normal control group (*p =* .0011 and *p = <*.0001, for oral and control groups, respectively) regarding the BG level and the percent reduction.

Despite the constant effect of the transdermal route after 6 h, the orally treated mice showed a significant decrease in the BG level compared to the 2 h level, as well as the transdermal treated group (*p =* .02). However, no significant difference in the BG at this period was recorded between the normal untreated animals and those receiving the CFZ NLCG. Nevertheless, after 20 h of treatment, no significant difference in BG was found between all groups.

These results are quite consistent with that observed in the above two test models, where the presence of food within the first 2 h can be responsible for the elevated BG level in the orally treated group, while the transdermal route was not affected postprandially. Of note, the drug absorption is known to be delayed in the presence of food. However, after 6 h and while the transdermal effect continues almost at the same pattern, the oral route decreased the BG significantly relative to its 2 h level. The data also showed significant difference in BG level between the transdermal and oral route. This effect can be owed as mentioned previously to its mutual effect on SGLT1 and the activation of the incretin peptides, besides the intact pancreatic activity of normoglycemic mice that reflects an increase in insulin secretion, such case was not available in the hyperglycemic mice in the OGTT as the contribution of the pancreatic activity was abolished.

##### 3.3.6.3. *In vivo* skin irritation test

The animals were examined for the presence of erythema and edema. The degree of erythema and edema were evaluated for calculation of SPI. The PII for the formalin aqueous solution was equal to 2.16, and the PII for the transdermal test formula was equal to 0.38. PII equal to or less than 2 is considered nonirritant according to (Draize et al. (1944), i.e. the formula has negligible irritation according to classification of irritation category.

## 4. Conclusion

Canagliflozin was efficiently loaded on the prepared NLCG systems with a concentration reaching 4%. CFZ-NLCG2 succeeded in overcoming challenges of skin barrier and delivering the drug transdermally at therapeutic levels that could exert an antihyperglycemic activity equivalent to or even exceeding that obtained in the oral route. The supremacy of the amphiphilic tri-block copolymer over conventional surfactants in the development of the LCG phase is highly declared in this study. This is in regard to its ability to form the largest gel phase area, high resilience to the effect of the added three types of co-solvents and in the preservation of the mesophase stability. This potentiated activity via the transdermal route would certainly allow further reduction in the drug therapeutic dose and maximizing the desired drug selectivity for renal SGLT and inhibiting any sort of selectivity for intestinal SGLT. In addition, the smart combination of a co-solvent to the prepared systems facilitated their formation at minimal effort; simple mixing technique, while minimizing the adherence of the formula to the wall of the container. Such behavior is a crucial criterion in industrial application and would consequently minimize the vast barrier between lab-based revolutionary research and its industrial implementation via upscaling. This performance would not only reduce the amount of loss but most importantly insures homogeneity and uniformity of contents in the batch formula. Thus, their presence should be considered as an integral part of the excipients system in the scaling up manufacturing process of such formulations.

The aforementioned outcomes would highly encourage performing further pharmacokinetic and pharmacodynamic evaluations of the transdermal CFZ-NLCG2 formulation on humans in the near future.

## Declarations of interest

None

## 5. Role of the funding source

This work has not been funded by any institution.

All experimental procedures were approved by the research ethics committee of Faculty of Pharmacy, Future University in Egypt (PT: REC-FPSPI-11/73).
